# Understanding the Utility of Automation for Diagnosing Spontaneous Bacterial Peritonitis and Its Variants

**DOI:** 10.7759/cureus.86782

**Published:** 2025-06-26

**Authors:** Yasha Mukim, C Ganesh Pai, Chaitanya Tellapragada, Vandana K.E

**Affiliations:** 1 Department of Microbiology, Employees’ State Insurance Corporation (ESIC) Medical College and Hospital, Faridabad, IND; 2 Department of Gastroenterology, Kasturba Medical College, Manipal, Manipal, IND; 3 Divison of Clinical Microbiology, Karolinska Institute, Stockholm, SWE; 4 Department of Microbiology, Kasturba Medical College, Manipal, Manipal, IND

**Keywords:** ascites, automated culture, enterobacterales, spontaneous bacterial peritonitis (sbp), turnaround time

## Abstract

Background: Spontaneous bacterial peritonitis (SBP) is the most frequent bacterial infection in cirrhotic patients with ascites, marking early hepatic decompensation. Variants of SBP are classified based on ascitic fluid polymorphonuclear neutrophil count and culture positivity.

Methods: A prospective cross-sectional study was conducted from 2014 to 2016 at a tertiary care hospital in South India. The study compared the diagnostic yield of conventional versus automated culture methods for detecting SBP and its variants. Ascitic fluid samples were cultured using conventional culture and/or automated culture methods, based on the clinician’s preference.

Results: Among 190 patients with ascites, automated culture was performed in 175 patients (92%) and conventional in 82 patients (43%). An automated blood culture system detected pathogens in 70 patients (40%), whereas conventional methods were positive in only eight cases (9.8%). After excluding contaminants, the overall culture positivity was seen in 45 patients (23.1%). *Escherichia coli* was the most frequently isolated pathogen. Notably, rare organisms such as *Campylobacter* spp., *Aeromonas* spp., *Beta-hemolytic* s*treptococci*, and *Salmonella typhimurium* were isolated exclusively via automated culture. Accordingly, patients were also categorized into SBP (24 patients; 13%), culture-negative neutrocytic ascites (CNNA) (17 patients; 9.2%), and monobacterial bacterascites (MNB) (18 patients; 9.7%).

Conclusion: Automated culture systems significantly outperform conventional methods in detecting bacterial pathogens in ascitic fluid, with a fourfold higher detection rate. Their ability to isolate fastidious organisms underscores their utility as the preferred first-line diagnostic tool in suspected SBP.

## Introduction

The development of ascites is typically the earliest clinical indicator of hepatic decompensation in individuals with cirrhosis. Among the various precipitating factors of acute decompensation, bacterial infections play a pivotal role and are closely associated with increased mortality in cirrhotic patients [1]. Evidence suggests that 30% to 50% of patients with cirrhosis are either infected at the time of hospital admission or acquire infections during the course of hospitalization. These infections significantly contribute to mortality, accounting for approximately one-quarter of all deaths in this patient cohort [2-4].

Spontaneous bacterial peritonitis (SBP) is the most frequent bacterial infection, followed by urinary tract infection, pneumonia, skin and soft tissue infections, and spontaneous bacteremia [5]. The prevalence of SBP in the past was relatively low at 5% to 10% in cirrhotic patients with ascites [6]. However, recent studies using newer diagnostic criteria and improved culture techniques have estimated a prevalence of 10% to 30% in cirrhotic patients with ascites admitted to hospitals and 3.5% among outpatients.

SBP is believed to arise from a multifactorial pathophysiological process intrinsic to patients with cirrhosis and ascites. Although several mechanisms have been implicated in its development, only a subset demonstrates a strong and consistent association. Key contributing factors include intestinal bacterial overgrowth, increased intestinal permeability, and bacterial translocation, along with dysfunction of the host immune response and impaired reticuloendothelial and ascitic fluid defense mechanisms. These elements act synergistically to compromise peritoneal immunity and facilitate infection [6].

Although identifying the pathogen plays a major role in the management, ascitic fluid cultures often show negative results in patients with clinical signs and symptoms of SBP. Therefore, ascitic fluid cellular analysis is the gold standard method for the diagnosis of SBP. The infection of the ascitic fluid is generally diagnosed based on an increased count of polymorphonuclear neutrophils in the ascitic fluid (>250cells/mm^3^), and the identification of the causal pathogen may not be given consideration because of the aforesaid reason.

Variants of SBP are based on the polymorphonuclear neutrophils in the ascitic fluid and its culture positivity: Classic SBP is where the polymorphonuclear cell count in the ascitic fluid is ≥250 cells/mm^3^ along with positive ascitic fluid culture; culture-negative neutrocytic ascites (CNNA) is where the ascitic fluid has a polymorphonuclear neutrophil count of ≥250 cells/mm^3^ but with negative ascitic fluid culture, and monomicrobial non-neutrocytic bacterascites (MNB) is where the polymorphonuclear neutrophil count is not elevated in the ascitic fluid but is culture positive.

Enteric gram-negative bacilli, especially *Escherichia coli*, are the most commonly encountered microorganisms, but gram-positive organisms such as *Streptococcus spp*., *Enterococcus spp*., or even *Pneumococcus spp. *can also be isolated [7]. When SBP is suspected but multiple organisms including anaerobes are recovered from the peritoneal fluid, the diagnosis must be reconsidered and a source of secondary peritonitis should be looked for.

Historically, Gram staining and conventional culture methods when used have shown poor diagnostic results in identifying the pathogen from the ascitic fluid. Conversely, using an automated blood culture system can aid better in isolating pathogens from the ascitic fluid.

This study aimed to evaluate and compare the diagnostic efficacy of conventional versus automated culture techniques for diagnosing SBP and its variants. Additionally, the role of concurrent blood cultures in enhancing diagnostic yield was assessed. The study also included an analysis of the antimicrobial susceptibility profiles of the isolated pathogens.

## Materials and methods

A prospective observational study was carried out at a tertiary care hospital in South India from August 2014 to April 2016. All suspected cases of SBP were included in this study. After obtaining informed consent from the patient, a minimum of 10ml of ascitic fluid was drawn using all aseptic precautions. The sample was either received in a sterile leak-proof container or was directly inoculated into a BacT/ALERT FA PLUS (Biomerieux, France) bottle, or both, depending on the clinician’s choice.

All the ascitic fluid received for conventional culture was centrifuged at 3000 rpm for 10 minutes. The pellet was cultured on 5% Sheep Blood agar and MacConkey’s agar and was inoculated into Tryptic soy broth along with the supernatant, and it was incubated for seven days. The day of turbidity was noted, and a smear was prepared for Gram’s Stain along with a subculture onto 5% sheep blood agar. In the absence of turbidity, a blind subculture on day 7 was performed onto a 5 % sheep blood Agar and was incubated.

All the specimens that were received for automated culture in the BacT/ALERT system were hourly monitored for positivity. When signaled positive, the bottle was withdrawn from the machine and was observed for color change as well as changes in the graph. The specimen, which signaled positive, was then cultured onto 5% sheep blood agar and MacConkey’s agar along with microscopy.

Blood culture was performed simultaneously in patients suspected of having ascitic fluid infection. Using aseptic precautions, a minimum of 10 ml of blood was drawn from two different sites at different time intervals and was inoculated into a set (two bottles) of BacT/ALERT FA PLUS (Biomerieux, France). Once inoculated, the bottles containing the sample were sent to the laboratory without any delay and were loaded into the automated blood culture system.

The culture plates were examined for any bacterial growth after 18-24 hours of incubation. If no growth was observed after 24 hours of incubation, the plates were further incubated for 72 hours. Growth on culture was further processed for identification and antimicrobial susceptibility testing. Identification was performed using VITEK MS v2.0 MALDI-TOF (BioMerieux Inc., France), and susceptibility was done on VITEK 2 AST (BioMerieux Inc., France).

## Results

A total of 190 patients presenting with ascites were recruited in the study from September 2014 to March 2016. The mean age of the study population was 50.7 ±14.8 years. The majority of the study subjects were male (160, 84.3%) with a male: female ratio of 5.3:1.

Culture positivity

**Figure 1:**
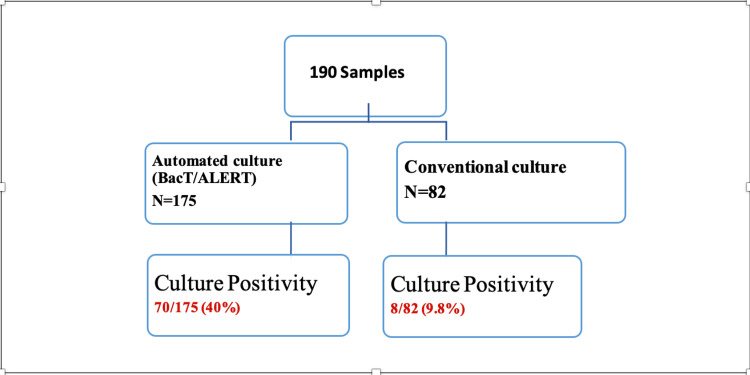
Flowchart showing total samples received and culture positivity

Automated culture using BacT ALERT was performed among 175 of the 190 patients (92%) and conventional culture was performed among 82 of the 190 (43%) study subjects. Microbiological culture using both conventional and automated culture systems was carried out among 67 (35.2%) of the study subjects. Culture positivity using BacT ALERT was observed among 70 of 175 (40%) patients and that using the conventional culture method was 8 of 82 (9.8%) patients as depicted in Figure 1.

Sensitivity and the negative predictive value of the conventional culture method deduced using the automated culture system as the reference method in the present study were 50 % (95%CI: 15-84) and 94.3% (95% CI: 86.2- 98.4), respectively.

Of the total 190 patients recruited in the study, results of automated culture from blood and ascitic fluid specimens were available for 124 patients. Among these 124 patients, 12 (10%) had the same pathogen isolated from both blood and ascitic fluid specimens.

**Figure 2:**
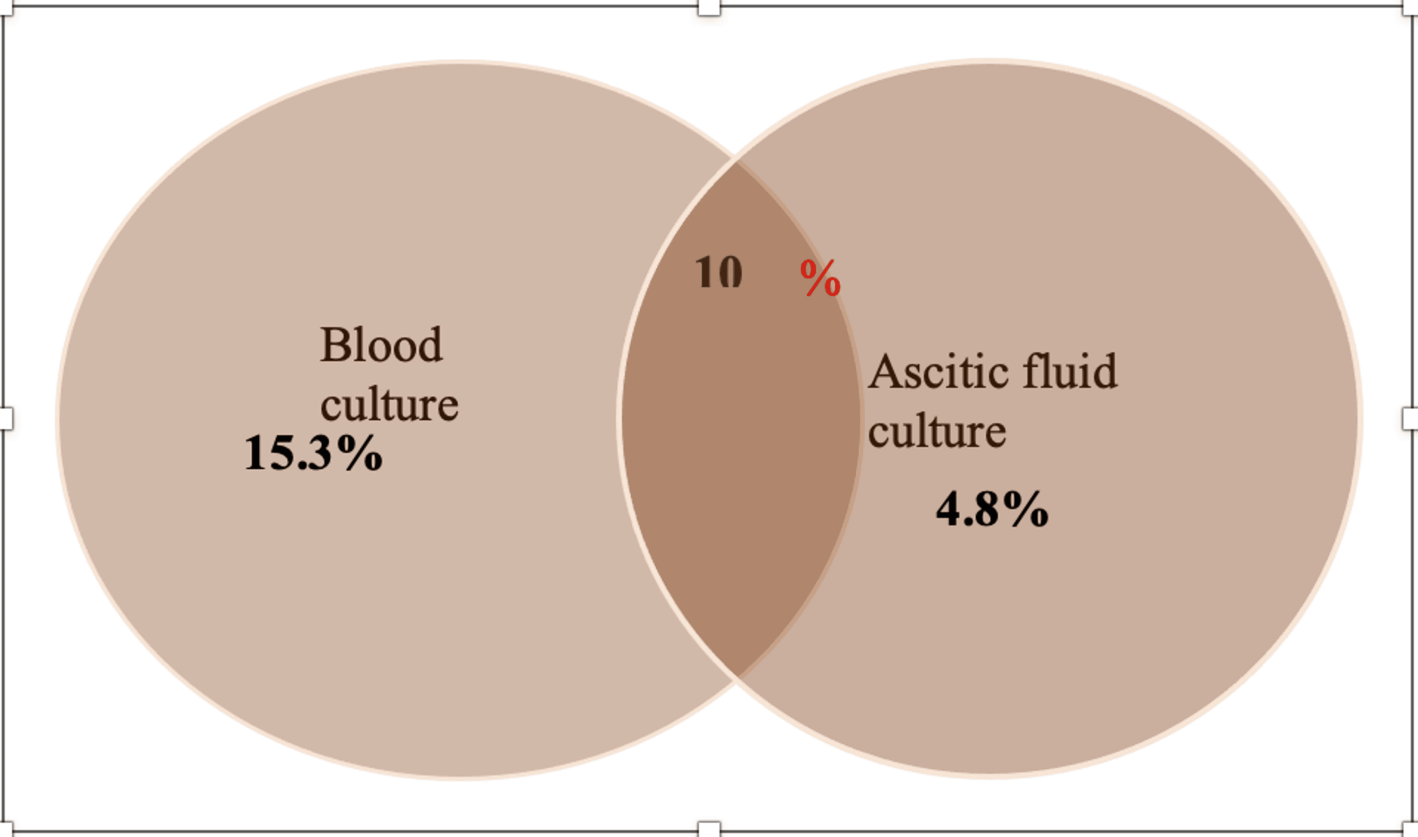
Pathogen isolated only in blood culture and ascitic fluid culture individually and in combination among 124 patients

Nineteen (15.3%) patients were positive for blood culture, but ascitic fluid culture remained negative. Those 19 cases where only blood culture was positive had either urine, wound, or bile as the source. Furthermore, there were six (4.8%) patients who had positive ascitic fluid culture, but blood culture remained negative. Correlation of culture positivity from blood and ascitic fluid specimens is depicted in Figure 2.

Laboratory diagnosis of ascitic fluid infection using absolute neutrophil count was achieved among 184 patients. Based on the absolute neutrophil count and microbiological culture report, the study subjects could be grouped as those with SBP (24 patients; 13%), CNNA among 17 patients (9.2%), and MNB among 18 patients (9.7%). 

While comparing the clinical outcome among these patients we found that clinical outcome holds significant in cases of MNB with a p-value of 0.003 as depicted in Table 1.

**Table 1:** Summary of the clinical outcome in terms of cure and worsening in SBP, CNNA, and MNB patients SBP: Spontaneous bacterial peritonitis; CNNA: culture-negative neutrocytic ascites; MNB: monomicrobial non-neutrocytic bacterascites

Infection Type	Cure (%)	Worsened (%)	Lost to Follow-up (%)	p-value
SBP (n = 24)	62.5%	20.8%	16.6%	0.640
CNNA (n = 17)	47.0%	11.7%	41.1%	0.315
MNB (n = 18)	27.7%	44.4%	27.7%	0.003

Bacterial etiology

Culture positivity for microbes was observed among 72 (37.8%) of the study specimens tested. Among the 72 patients with positive microbial culture, 45 (62.5%) patients had significant pathogens in their ascitic fluid and the other 27 (37.5%) patients had their ascitic fluid culture yielding skin contaminants. Thus, the overall culture positivity for bacterial pathogens from ascitic fluid specimens in the present study was 23.1% (n=45), after excluding the skin contaminants.

From the 45 patients with a positive pathogen culture, a total of 52 bacteria were isolated and identified. Gram-negative bacteria (37/52; 71%) were the leading pathogens in the present study. Among the Gram-negative bacteria, bacterial species belonging to family Enterobacteraceae (30, 57%) were most commonly isolated. *Enterococcus spp*. (9, 17.3%) was more commonly isolated among the Gram-positive organisms. Other organisms isolated were *Beta haemolytic Streptococci *(5, 9.6%), followed by* Acinetobacter spp*. (2, 3.84%), *Streptococcus pneumonia* (1, 1.9%), *Campylobacter spp*. (1, 1.9%), *Staphylococcus aureus* (1, 1.9%), *Aeromonas spp*. (1, 1.9%), *Stenotrophomonas maltophilia* (1, 1.9%), *Elizabethkingia meningoseptica* (1, 1.9%), and non-fermenter gram-negative bacilli (1, 1.9%), listed in Table 2.

**Table 2:** Frequency and percentage of organisms isolated from the ascitic fluid (N = 52) GNB: Gram-negative bacilli

Organism	Frequency (out of 52)	Percentage (%)
Gram Negatives		
Escherichia coli	21	40.0
Klebsiella pneumoniae	8	15.3
Acinetobacter spp.	2	3.8
Salmonella typhimurium	1	1.9
Stenotrophomonas maltophilia	1	1.9
Non-fermenter GNB	1	1.9
Elizabethkingia meningoseptica	1	1.9
Aeromonas spp.	1	1.9
Campylobacter spp.	1	1.9
Gram Positives		
Enterococcus spp.	9	17.3
Beta haemolytic streptococci	5	9.6
Staphylococcus aureus	1	1.9
Streptococcus pneumoniae	1	1.9

Antimicrobial resistance

**Figure 3:**
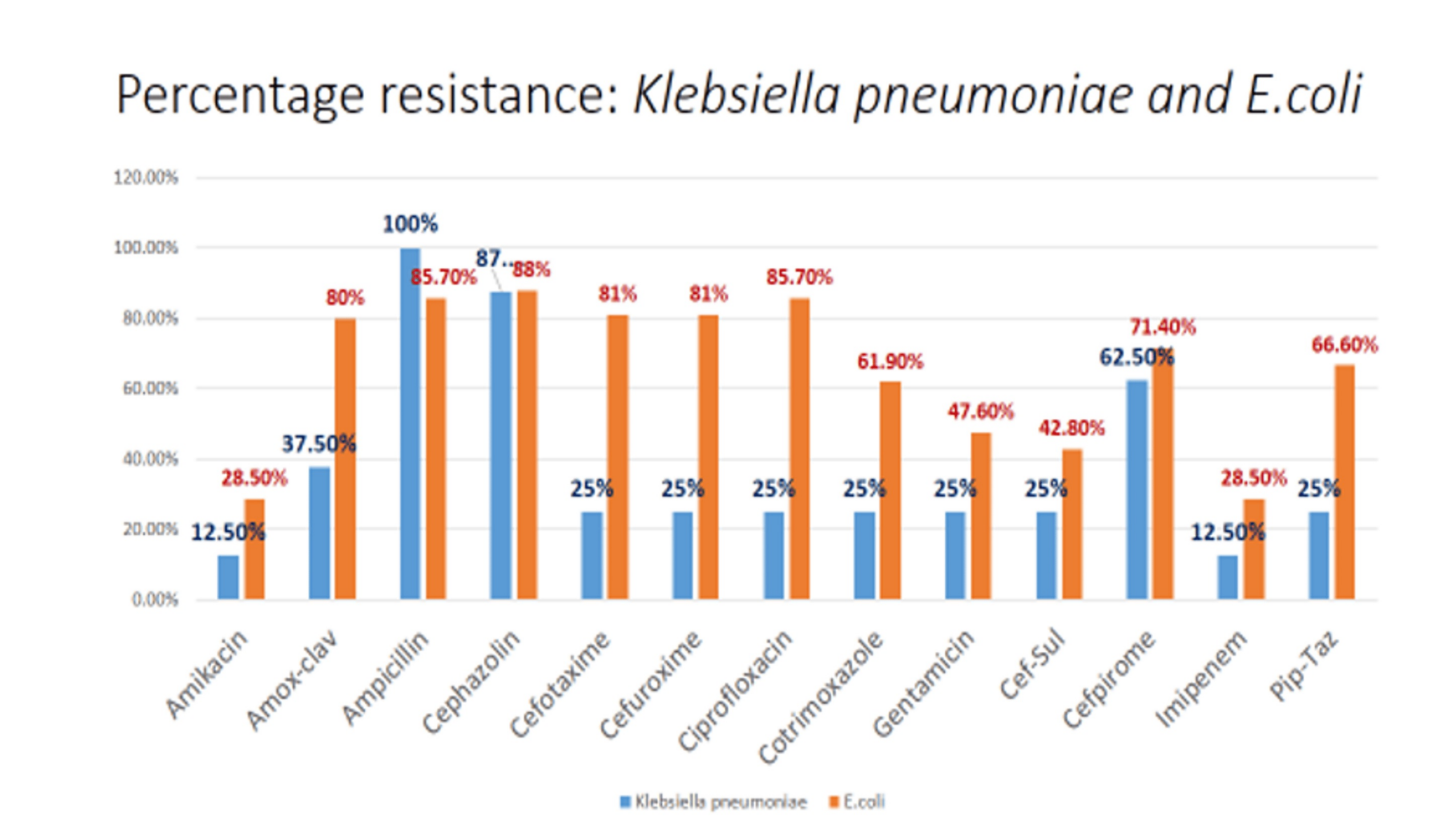
% Resistance among Enterobacterales

Antimicrobial susceptibility patterns in terms of percentage resistance to commonly tested antibiotics among *E. coli *and *K. pneumoniae* isolates are depicted in Figure 3.

**Figure 4:**
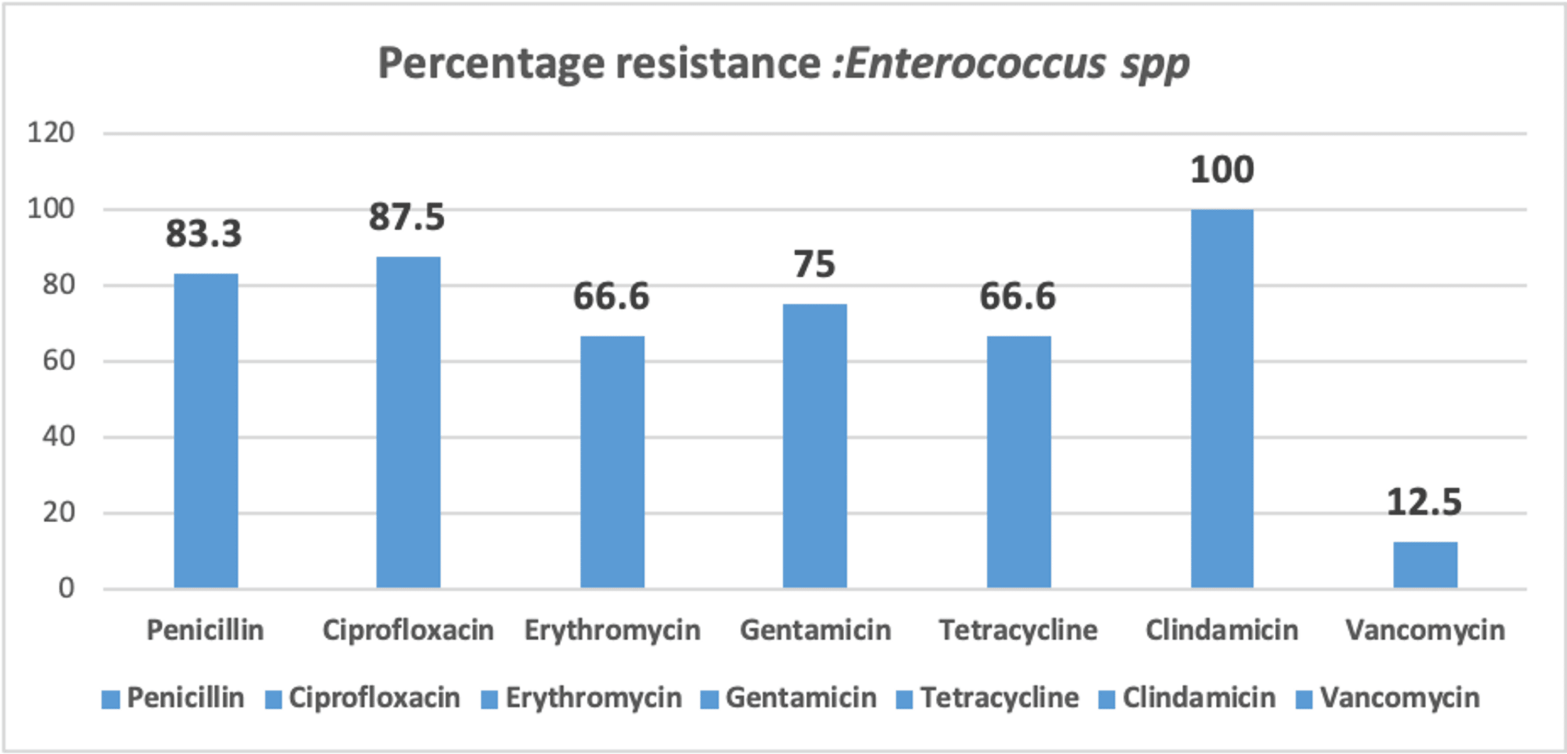
% Resistance among Enterococcus spp.

Antimicrobial susceptibility patterns among *Enterococcus spp. i*solated in the present study is depicted in Figure 4.

## Discussion

Culture positivity

Overall culture positivity for bacterial pathogens in this study from non-repeat ascitic fluid specimens was 23.1%, after excluding the skin contaminants. A similar study by Kamani et al. studied 187 patients of SBP and CNNA and found out the culture positivity for SBP to be 23.5% although they performed bedside inoculation of the sample into aerobic and anaerobic blood culture bottles (Bactec 9240) [8]. Another study by Oladimeji et al. elaborates culture positivity rates of SBP and CNNA as 67.7% and 33.3% in their study, although their sample size was too small compared to this study [9]. Both the studies recruited patients with a background of ongoing cirrhosis of the liver.

An Indian study stated Gram-negative culture positivity of up to 79.6% among patients presenting with surgical peritonitis, which was different from our group of patients [10].

While evaluating the course of culture positivity, we found that the earlier rate of pathogen detection varied from time to time. Runyon et al., in their study performed in 1984, reported culture positivity rates of 42% using a conventional culture technique but observed an increase in the culture positivity rate to 91% while inoculating into blood culture bottles [11].

The lower rates of culture positivity in the past could be attributed to the low bacterial concentration in the ascitic fluid and also to the culture techniques used. With the advancement in time and diagnostic techniques, bedside inoculation of the ascitic fluid into an automated blood culture system has increased the rate of pathogen detection on a large scale.

Ascitic fluid infections mainly have three subtypes: the prototype being SBP. SBP is defined as a positive culture with ≥250 polymorphonuclear cell count, whereas CNNA is defined as ≥250 polymorphonuclear cell count with a negative ascitic fluid culture. This could be attributed to the poor culture techniques and prior antibiotics or low opsonic activity of the ascitic ﬂuid. Another type, MNB, is defined as <250 polymorphonuclear cell count with a positive ascitic fluid culture. It is believed that this entity may resolve spontaneously or progress to SBP and has similar mortality rates compared to SBP and should be managed in the same way. Ascitic fluid infections were diagnosed in 70 patients (11.6 %) in a similar study. Of these, 57.1 % had CNNA, 35.8% had SBP, and 7 % had MNB. These findings are very similar to the findings of our study [12].

Bacterial aetiology

In this study, we observed the predominance of Gram-negative organisms, which accounted for 71% among the patients. Similar rates have been reported (80%) by Fernández et al. in their study, which included 138 suspected cases of SBP in the year 2002 [13]. Another study by Runyon et al. in 1988 also showed predominance of enteric Gram-negative organisms of up to 60% when isolated from ascitic fluid samples [14].

Among the Gram-negative bacteria, species belonging to the family Enterobacteriaceae (57%) were most commonly isolated. Among the enteric Gram-negative organisms, *E. coli *(70%) was most commonly isolated from the ascitic fluid samples. Predominance of this organism over others has been stated in many other studies. In one such study, which compiled data from 1971-1991, reported *E. coli* to be the most commonly isolated organism, accounting for almost 46% [15]. Later studies by Conn et al. [16] and Kerr et al. [17] also reported *E. coli *to be the most common pathogen isolated from the ascitic fluid, with an isolation rate of 66% and 72%. A recent study by Oladimeji et al. conducted in 2010 stated a similar isolation pattern of *E. coli* (70%), which was comparable to our study [9].

This predominance of enteric Gram-negative organisms particularly *E. coli *causing SBP can be attributed to the fact that it forms a major part of the gut flora. With intestinal hypomotility and lowered local immunity, there is an overgrowth of the organism in the gut, giving it an opportunity to cross the barrier and enter a sterile fluid, causing infection.

Gram-positive organisms were also isolated from the ascitic fluid samples almost accounting for 29%. Amongst the Gram-positive organisms, *Enterococcus spp*. was most commonly isolated (17.3%). Many studies have reported isolation of Gram-positive organisms from ascitic fluid cultures, but at a rate of <25% [18,19]. An observational study among cirrhotic patients with SBP reported predominance of Gram-positive organisms such as CoNS from ascitic fluid cultures, accounting for almost 27% which was similar to our study [20]. Although CoNS are skin flora and usually reported as skin contaminants, in this study also it was also not taken into consideration in terms of treatment. Although with isolation of CoNS, a properly collected repeat culture should be requested when in clinical suspicion of SBP.

Isolation of Gram-positive organisms from ascitic fluid culture can be attributed to therapeutic interventions and chronic antibiotic usage. Other than SBP, these patients are also prone to acquiring pneumonia and urinary tract infections, which are usually caused by Gram-positive organisms like *Streptococcus pneumoniae*, *Enterococcus spp*., *Streptococcus spp.,* and *Staphylococcus aureus* [13].

In this study, rare organisms were also isolated, like *Campylobacter spp*., *Aeromonas spp*., *E. meningoseptica*, and *S. maltophilia*. Although the cell count was high in the patients where *Campylobacter spp*. and *Aeromonas spp*. were isolated, both the groups had clinical cure.

Cell count was <250 cells/mm^3^ in cases where* E. meningoseptica *and *S. maltophilia* were isolated. Mortality was observed where *E. meningoseptica* was isolated, which could also be attributed to the polymicrobial event in this case, and also to the resistant nature of the organism, which was isolated.

*Aeromonas spp*. and *Campylobacter spp*. can cause ascitic fluid infections and could be attributed to the bacterial translocation from the gut in these patients. Frequent Instrumentation and repeated puncture can be a cause for isolation of other rare organisms from the ascitic fluid, and clinical acumen should be sought for in such cases.

Antibiotic resistance

Almost 76.8% of patients in this study received empiric antibiotic therapy during their course of admission. Ceftriaxone was the most commonly administered empiric drug (48.9%).

Maximum resistance observed amongst the commonly isolated organisms was to ampicillin (85.7%), ciprofloxacin (85.7%), and cefotaxime (81%), whereas the least resistance was to amikacin (28.5%), meropenem (28.5%), and cefoperazone-sulbactam (42.8%).

Third-generation cephalosporins (cefotaxime/ceftriaxone) remain the cornerstone of treating SBP. But with the increase in resistance pattern to the third-generation cephalosporins observed in our setup, revision of empirical therapy may be needed. A similar study was done, which suggested a change in empirical therapy from ceftriaxone to cefoperazone-sulbactam, due to high resistance to third-generation cephalosporins [12].

Although this study focused on comparing the utility of automated versus conventional culture systems, the inclusion of molecular diagnostic methods could have provided additional depth and clinical relevance. Future studies are planned to incorporate molecular techniques to enhance the comprehensiveness of the findings.

## Conclusions

SBP was the most prevalent ascitic fluid infection in this study, followed by MNB and CNNA. Gram-negative bacteria were the predominant pathogens, with *E. coli* being the most frequently isolated organism. The automated culture system demonstrated a significantly higher culture positivity rate compared to conventional methods, emphasizing its superiority as a first-line diagnostic tool. Furthermore, the automated system was able to detect fastidious organisms that were not identified by conventional culture techniques, highlighting its added diagnostic value in clinical practice.
